# Soil Moisture and Fungi Affect Seed Survival in California Grassland Annual Plants

**DOI:** 10.1371/journal.pone.0039083

**Published:** 2012-06-12

**Authors:** Erin A. Mordecai

**Affiliations:** Department of Ecology, Evolution, and Marine Biology, University of California, Santa Barbara, Santa Barbara, California, United States of America; Argonne National Laboratory, United States of America

## Abstract

Survival of seeds in the seed bank is important for the population dynamics of many plant species, yet the environmental factors that control seed survival at a landscape level remain poorly understood. These factors may include soil moisture, vegetation cover, soil type, and soil pathogens. Because many soil fungi respond to moisture and host species, fungi may mediate environmental drivers of seed survival. Here, I measure patterns of seed survival in California annual grassland plants across 15 species in three experiments. First, I surveyed seed survival for eight species at 18 grasslands and coastal sage scrub sites ranging across coastal and inland Santa Barbara County, California. Species differed in seed survival, and soil moisture and geographic location had the strongest influence on survival. Grasslands had higher survival than coastal sage scrub sites for some species. Second, I used a fungicide addition and exotic grass thatch removal experiment in the field to tease apart the relative impact of fungi, thatch, and their interaction in an invaded grassland. Seed survival was lower in the winter (wet season) than in the summer (dry season), but fungicide improved winter survival. Seed survival varied between species but did not depend on thatch. Third, I manipulated water and fungicide in the laboratory to directly examine the relationship between water, fungi, and survival. Seed survival declined from dry to single watered to continuously watered treatments. Fungicide slightly improved seed survival when seeds were watered once but not continually. Together, these experiments demonstrate an important role of soil moisture, potentially mediated by fungal pathogens, in driving seed survival.

## Introduction

Most plant life cycles begin with seeds: to complete the life cycle, seeds must germinate, survive, mature, and produce new seeds. The environmental factors that control the beginning of the life cycle—seed survival in the soil and germination—are often overlooked in ecological studies. Yet many important processes that affect plant population dynamics and community composition depend on seed survival and germination [1]. For example, seed survival in the soil can buffer plant populations against environmental variability [2], [3]. Seed survival and germination cues are important mechanisms by which plants respond to environmental fluctuations. Germination cues often correspond with the environmental conditions most favorable for seedling growth, survival, and/or avoidance of competition [4]. Seed banking, the storage of ungerminated seed in the soil between growing seasons, can buffer population losses due to competition or poor growing conditions. In variable climates such as coastal California, seed survival and germination vary substantially between years, affecting both the dynamics of individual populations and the outcome of competition between species [5].

Understanding the influence of environmental conditions on seed survival and germination is critical not only for population and community ecology but also for restoration and conservation [6]. Removing unwanted species requires understanding the seed bank and controls over its longevity. Conversely, for native species that are the target of restoration efforts, sites and species with long-lived seeds may not need to be sown. Thus, understanding how environmental characteristics influence seed survival will clarify the positive and negative effects of seed banks on conservation and restoration.

Fungal pathogens reduce seed survival in many habitats [7]–[13]. Soil fungi respond to moisture, plant litter, and other soil characteristics [10], [14]. Although soil fungi are ubiquitous, their impact on survival depends on the combination of fungus and seed species [11], [15]. Soil fungi can kill seeds, persist as harmless commensals, or even protect seeds [13]. Of the fungal pathogens, some are generalists while others are closely associated with particular species of seeds or plants in the surrounding community [16]. Pathogens may force a race for survival between the seed and the fungus [17], generating selective pressure that influences the timing of germination. Because of these properties, some soil pathogens may be useful as microbial biocontrol agents for weed seed banks [18]. Because ungerminated seeds are not yet dependent on nutrients and water, much of the influence of the environment on seed survival may be mediated by fungal pathogens, for example if wet conditions promote fungal pathogens that kill seeds.

In light of the importance of seed dynamics for a range of ecological applications, it is important to understand variability in seed survival in the soil across species, space, and time, and how this variation depends on abiotic and biotic factors. In particular, controls over seed survival at a landscape level are not well understood. To address this gap, I conducted a one-year seed survival survey across eight annual plant species in Santa Barbara County, California. The region has a Mediterranean climate, and rainfall varies substantially in quantity and timing between years [5]. Seed survival was measured at 18 sites that differ in vegetation cover (grassland or coastal sage scrub), proximity to the coast (inland or coastal), and soil moisture. To test the extent to which soil fungi drove the results from this field survey, I conducted two experiments that manipulated environmental conditions and fungi. California grasslands are heavily invaded by annual grasses that deposit thick layers of thatch, which may modify soil microclimate, promote fungal growth, or inoculate seeds on the ground—all of which could influence fungal attack on seeds. Therefore, I first measured seasonal variation in responses to exotic annual grass cover and soil fungi by removing grass litter and adding fungicide across winter and summer seasons for six species. I tested for effects of thatch removal and interactions between thatch and fungicide. Second, to examine the relationship between soil moisture and fungal pathogens more directly, I applied watering and fungicide treatments in the laboratory for seven annual plant species.

I hypothesized that seed survival would be lower in wetter environments and in the winter (the rainy season) as opposed to the summer, due in part to increased fungal growth in moist soil. I also expected exotic grass thatch to influence seed survival by modifying soil microclimate and thereby promoting fungal growth. Because seed species differ in size, seed coat, shape, phylogenetic origin, and other factors, I expected the species to vary substantially both in seed survival and in their responses to environmental drivers.

## Methods

### Field survey of seed survival

To survey seed survival across geographic location and plant community type, I buried mesh bags containing seeds of eight species at 18 sites in Santa Barbara County, California. The climate is Mediterranean with a strong coastal influence. Winters are cool and wet and summers and are hot and dry. Temperatures are more moderate year-round in the coastal sites due to low-hanging clouds and fog occurring throughout the summer. The inland sites are hotter and drier in the summer. Annual precipitation is highly variable, usually coming in a series of heavy rainstorms from December through March.

The species used in the experiment are a mixture of native and exotic annual grasses and forbs that occur in southern California grasslands (Table 1). The seeds used in this survey were purchased from S & S Seed Company in Carpinteria, California, and all genotypes were from Santa Barbara County. Each seed bag consisted of a mesh strip with a separate compartment for each species, each containing 50 seeds. The compartments were 7.5 cm wide by 10 cm tall with a 2.5 cm strip separating each compartment from the next, to prevent contamination across species. Seed bags were buried on December 14, 2008, prior to the first rain of the season, and recovered on October 24, 2009, at the end of the growing season and prior to the onset of the next season's rain.

**Table 1:** Description of species used in the study.

Species	Experiment used	Plant type	Provenance
*Clarkia purpurea*	field survey	Forb	Native
*Lupinus bicolor*	field survey	Forb	Native
*Hemizonia fasciculate*	field survey	Forb	Native
*Amsinkia intermedia*	field survey	Forb	Native
*Phacelia distans*	field survey	Forb	Native
*Brassica nigra*	field survey	Forb	Exotic
*Erodium cicutarium*	field survey	Forb	Exotic
*Vulpia microstachys*	field survey, field experiment, lab experiment	Grass	Native
*Plantago erecta*	field experiment, lab experiment	Forb	Native
*Chaenactis glabriuscula*	field experiment, lab experiment	Forb	Native
*Salvia columbariae*	field experiment, lab experiment	Forb	Native
*Chorizanthe palmerii*	field experiment, lab experiment	Forb	Native
*Avena barbata*	field experiment, lab experiment	Grass	Exotic
*Hordeum vulgare*	field experiment, lab experiment	Grass	Exotic
*Bromus hordeaceus*	lab experiment	Grass	Exotic

Native species are native to California grasslands; exotic species are widespread invaders from Europe. All species are annuals. For brevity, throughout the paper I refer to the species by their genus only.

The sites in the study represent a mixture of coastal and inland geography and grassland and coastal sage scrub (CSS) vegetation. Coastal sites were located in the region of Santa Barbara County between the coastal mountain range and the ocean, while inland sites were located north (inland) of the coastal range. Grassland sites were dominated by exotic grasses and contained few shrubs. Coastal sage scrub sites (characterized by vegetation, not geographic location), contained mostly sagebrush, sage, and other shrubs, along with a few understory grasses and forbs. There were a total of five coastal CSS, four coastal grassland, five inland CSS, and four inland grassland sites (sample sizes were uneven due to loss of several of the original sites). All necessary permits were obtained for this and the following field experiment. Ten seed bags were buried at each site within a ten-meter area, for a total of 180 bags. At least 152 intact samples were recovered for each species.

I measured soil moisture on March 18, 2009, in the middle of the growing season at approximately peak soil moisture but not within two weeks of a rain event. At each site I collected two soil cores and measured soil moisture by weight, comparing wet weight of soil to dry weight after 3 days in the drying oven at 60°C. I calculated percent soil moisture from each sample gravimetrically, and averaged the two values for each site.

Due to the large number of seeds in the study, full seed viability analysis was not feasible. Instead, I treated the number of seeds that germinated either in the field or in the lab as a proxy for survival. The correlation between germination and survival was 0.95 in a laboratory experiment in which I tested both (discussed below), suggesting that germination in the laboratory is a good proxy for seed survival. To assess germination I cut open the bags and placed them on wetted germination paper (Versa-Pak, Anchor Paper Company) in clear plastic germination boxes. The seeds were held at 15°C for five days, then placed on the lab bench at room temperature for three weeks [5]. Germinated seeds were counted and removed daily.

I analyzed the effects of vegetation type, geographic location, and soil moisture on seed germinability using binomial generalized linear mixed models (GLMMs). The fraction of seeds that germinated out of the total number of seeds in each bag was the response variable, so that each bag produced a single data point per species [19]. The basic model structure is:
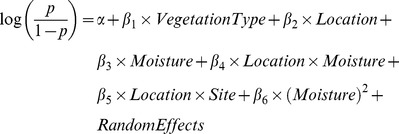
(1)where the left-hand side is the logit-transformation of *p*, the proportion of seeds that germinated from each bag. *α* is the intercept, or the average response at the baseline condition, and the *β*'s represent the effect of each environmental variable relative to the baseline value. For example, in the models presented in Table 2 the baseline condition is coastal location, CSS vegetation, and soil moisture equal to the average across sites. *β_1_* represents the effect of grasslands, *β_2_* the effect of inland location, *β_3_* the effect of deviations from mean moisture, *β_4_* the interactive effect of deviations in moisture at inland sites, *β_5_* the interactive effect of inland grasslands, and *β_6_* the squared effect of deviations in mean soil moisture. Moisture squared was included to test for unimodal effects of soil moisture on seed survival. Deviations in soil moisture were used (i.e. the mean was subtracted), rather than absolute values, to improve model fitting. The categorical variables are coded as 0 for the baseline value and 1 for alternative values, so that for non-baseline values the model includes the intercept and the additional coefficient.

**Table 2:** Estimates of the regression parameters from the best fit models for the field survey.

	intercept		inland		moisture		grassland		inland×moisture		(moisture)^2^	
All species	−1.95	***	0.18		−0.48		0.176	.	−14.3	***	-	
Clarkia	−0.27		0.071		−2.14		-		−42.83	***	-	
Lupinus	−3.74	***	0.74	***	-		-		-		-	
Hemizonia	−1.09	***	−0.09		0.943		0.592	***	−21.14	***	-	
Amsinkia	−2.02	***	−0.21		−1.99		0.299	*	−10.75	**	-	
Brassica	−1.92	***	-		−0.63		-		-		−207.5	***
Vulpia	−2.2	***	−0.11		4.347	*	0.282	*	−25.91	***	-	
Phacelia	−0.99	***	-		-		-		-		-	
Erodium	−2.95	***	0.843	***	−7.56	**	-		-		-	

Significance codes: 0 ‘***’ 0.001 ‘**’ 0.01 ‘*’ 0.05 ‘.’ 0.1 ‘ ’ 1.

The intercept (*α* from equation 1) represents the baseline scenario of coastal location, CSS vegetation, and average soil moisture. The subsequent coefficients (*β*'s from equation 1) represent change in the intercept relative to the baseline. The moisture coefficients represent responses to deviations from the mean value. The first row lists the estimates of fixed effects from the best fit GLMM for all species combined. The subsequent rows list the best GLMM for each species separately. The dash indicates a factor that was not included in the best-fit model.

I first examined trends across species by analyzing all data together with species as a random effect in a mixed model. I then fit models to each species separately to pinpoint how species differed in their responses. To avoid pseudoreplication and control for the nested structure, I fit GLMMs with random effects of bag nested within site. This accounts for the fact that seed germination was measured at the bag level (i.e. ten bags per site) but predictor variables were measured at the site level. Including bag-level random effects also controls for overdispersion, i.e. higher variance than expected for binomial data [20]. I compared nested models containing subsets of the fixed effects using Akaike Information Criterion (AIC). In order to isolate differences among species, I fit GLMMs with the same fixed effects and nested random effects for each species separately. I selected the best model for each species using AIC. All models were implemented in R (R Core Development Team; r-project.org, version 2.11.1) using “glmer” in the “lme4” package.

### Field experiment on the effects of fungi and thatch

To measure the effect of fungi and exotic grass thatch on seed survival, I added fungicide and removed thatch over buried seed bags in Sedgwick Reserve in Santa Ynez, California. The site, located inland in Santa Barbara County on Figueroa Mountain, was one of the sites included in the field seed survival survey. The fungicide and thatch experiment was conducted in an invaded serpentine grassland with mixed forbs. Before the annual grass invasion, the area was probably dominated by native perennial bunchgrasses and annual forbs. Because exotic annual grasses create much thicker layers of dead plant material (thatch) than the native plants, and thatch modifies the soil microclimate potentially to the benefit of soil pathogens, I expected thatch to mediate seed survival.

I buried 64 seed bags in the grassland. Seed bags were constructed as described in the field survey, except that each contained 20 seeds of each species (listed in Table 1). Because this experiment focused on a single study site, I chose a set of species appropriate to this specific site. All seeds were collected from the site. The seed bags received factorial combinations of thatch absent/present and fungicide added/not added, for a total of 16 replicates per treatment combination. Following burial, thatch absent treatments were left with bare soil, while thatch present treatments had the displaced thatch replaced. Because this thatch was no longer rooted into the soil, these conditions may differ from naturally undisturbed thatch. I applied fungicide to seeds designated to that treatment by placing seeds in the seed bags after shaking them in vials with powdered Captan fungicide (Southern Ag).

I conducted the seed bag study once in the summer and fall, and again in the winter and spring. The summer seed bags were buried on July 9, 2008 and retrieved on October 28, 2008, before any rain had fallen. The winter seed bags were buried on December 12, 2008 and retrieved on July 28, 2009. Following retrieval, seeds were removed from seed bags and tested for germinability as described in the preceding section.

To examine general patterns of seed survival I fit binomial GLMMs with season, fungicide, and thatch as fixed effects and species and bag as random effects. The fraction of seeds in each bag that germinated was the response variable. The models follow the same basic structure as equation (1) for the field survival survey. I tested for season-by-fungicide and thatch-by-fungicide interactions. The bag-level random effect was included to correct for overdispersion. I compared nested models using AIC.

To compare responses between species, I removed the random effects and fitting generalized linear models (GLMs) for each species using the same fixed effects. It was not necessary to fit bag-level random effects for each species (as in the field survey) because the treatments were applied individually to each bag, and thus the experiment was not pseudoreplicated. To account for overdispersion I used quasi-binomial GLMs, which fit an additional dispersion parameter, c_hat_ [21]. Because AIC is not defined for quasi-binomial models, I compared model fits by quasi-AIC (qAIC). qAIC=(residual deviance/c_hat_)+2k, where c_hat_ is the dispersion parameter on the most complex model and k is one plus the number of parameters estimated [21]. GLMs were implemented in R using “glm” in the “stats” package.

### Laboratory experiment on the effects of water and fungicide

I further explored the relationship between soil moisture, fungi, and seed survival by manipulating fungicide and water in a laboratory experiment. I constructed mesocosms with seeds buried in field-collected soil, and applied fungicide to half of the samples following the methods described above. Each sample was either never watered, watered once at the beginning of the experiment, or watered twice per week throughout the experiment. Although percent soil moisture was not measured in this experiment, the samples that were never watered or watered only once were very dry at the end of the experiment, similar to conditions in the field during the summer drought period. Continuously watered samples remained moist throughout the study, with conditions similar to field soils during the rainy season in the winter and spring. Fungicide treatments were crossed with watering treatments for six treatment combinations. I used all the species from the field experiment plus one additional exotic grass, which was not available during the field experiment (Table 1).

The soil used in the experiment was collected from Sedgwick Reserve at the site of the field experiment. The soil was homogenized and sieved to 4 mm. Twenty seeds of a single species were sandwiched between two layers of mesh and buried within soil in a 6-cm diameter aluminum weighing boat. There were six replicates of each treatment for each species. The replicates were placed on trays in a growth chamber set to cycle between 11°C, 22°C, and 31°C daily with a 12-hour light-dark cycle. The experiment ran from August to December 2008.

At the end of the experiment, the seeds were removed and germinated as described above. Seeds that did not germinate after six days were treated with 350 ml of a 400 ppm gibberellic acid solution overnight to stimulate germination [5]. To test for viability of ungerminated seed, those that did not germinate were cut in half and soaked in 1% tetrazolium solution for one hour, staining live embryo pink. The tetrazolium treatment effectively allowed me to distinguish between live and dead seeds based on stained or unstained embryo, respectively. The number of viable seeds is equal to the number of seeds that germinated naturally or with gibberellic acid plus the number that stained with tetrazolium. I used the fraction of viable seeds in each replicate at the end of the experiment as the response variable in the analyses.

To evaluate the influence of the treatments on seed survival, I followed the data analysis methods outlined under the field experiment, with fungicide, water treatment, and their interaction as fixed effects and species and experimental block (tray) as random effects. I also fit quasi-binomial GLMs to each species individually.

## Results

**Figure 1:**
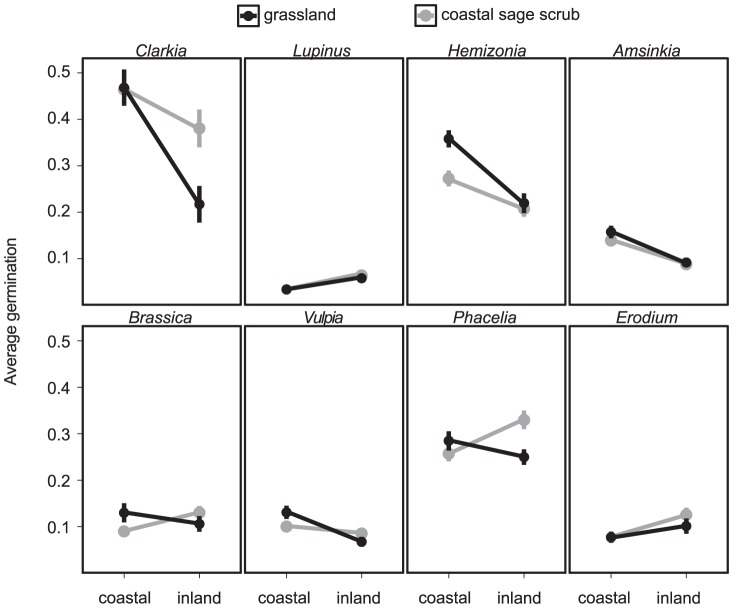
Average germination fraction by species, from the field survey. Different points represent each combination of location (coastal vs. inland) and vegetation type (coastal sage scrub, gray points vs. grassland, black points) for each species. Error bars are ±1 standard error.

**Figure 2:**
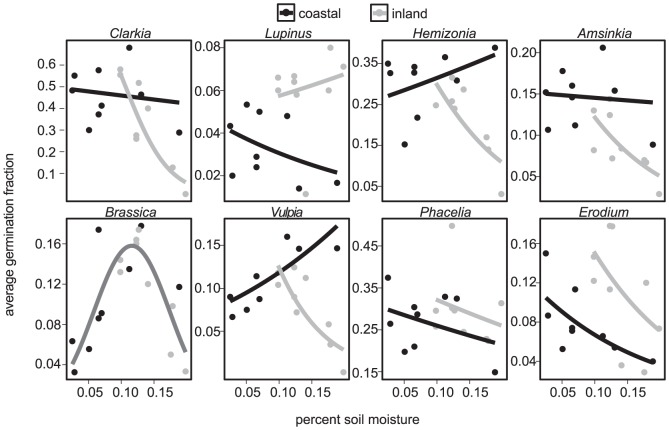
Average germination fraction by species as a function of soil moisture, from the field survey. Points are the average germination fraction pooled by site. Black points are coastal sites and gray points are inland sites. Lines are the fitted soil moisture by location models for each species except *Brassica*, which shows the (moisture)^2^ model because it was a better fit.

Water was an important predictor of seed survival across all three parts of the study. In the field survey, inland vs. coastal location interacted with soil moisture to affect seed survival (Figures 1, 2; Table 2). Overall, soil moisture reduced survival, but this decline was steeper inland than in coastal sites (Figure 2; Table 2, All Species model). Grasslands had slightly higher survival than coastal sage scrub sites (Figure 1; Table 2), suggesting that thatch may improve survival to some degree.

Soil moisture affected seed survival in the survey for all species individually except *Phacelia* and *Lupinus* (Table 2). Survival declined with moisture at inland sites for all the remaining species, but declined less steeply or even increased with moisture at coastal sites (Figure 2). *Brassica* survival peaked at intermediate moisture (Figure 2). These results suggest that seed responses to soil moisture are mediated by other unmeasured factors that vary geographically, such as soil type or other climate variables. Geographic location itself (independent of soil moisture) influenced survival for some species: *Lupinus* and *Erodium* had higher survival inland while *Clarkia, Hemizonia,* and *Amsinkia* had higher survival near the coast (Figure 1).

**Figure 3:**
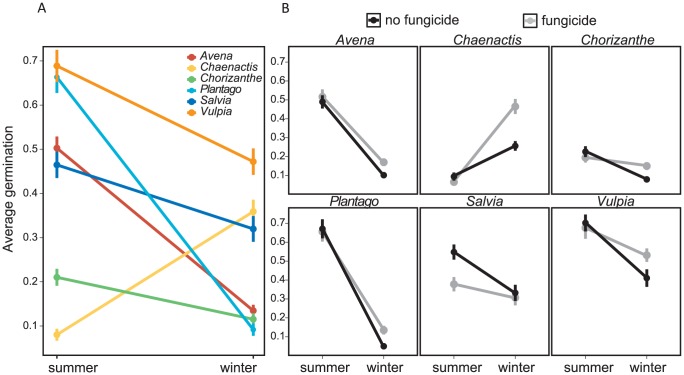
Average germination fraction from summer or winter with or without fungicide, from the field experiment. Differences in germination between summer and winter for all species (with both fungicide treatments combined), (a), plotted by fungicide treatment for each species separately, (b). In (b), black dots are treatments with fungicide, and gray dots are without fungicide. Error bars are ±1 standard error.

Some of the environmental drivers that may underlie the results of the field survey were highlighted in the field experiment. First, seed survival was lower in the winter than in the summer, a pattern which held for all species except *Chaenactis* (Figure 3; Table 3). Because winter is the rainy season and summer had no precipitation, this again suggests that soil moisture influenced seed survival. Fungicide improved winter survival but did not affect survival in the summer (Figure 3; Table 3). This effect was significant for *Chaenactis*, *Chorizanthe*, and *Plantago*, and the pattern also held for *Avena* and *Vulpia*. Fungi therefore contribute to over-winter mortality of seeds, and may be partly responsible for the decline in seed survival with soil moisture in the field survey. Although grasslands had higher survival than coastal sage scrub in the field survey, removal of grass thatch did not significantly affect survival in the field experiment (Table 3). This may be because thatch was no longer rooted into the soil, because thatch had already altered the soil biota and microclimate prior to removal, or because thatch does not strongly affect seed survival.

**Table 3:** Estimates of regression parameters from the best fit models for the field experiment.

	intercept		thatch	winter		no fungicide		winter×no fungicide	
All species	−0.5		-	−0.69	***	0.205		−0.87	***
*Avena*	0.011		*-*	−1.87	***	-		-	
*Chaenactis*	−2.66	***	*-*	2.52	***	0.399		−1.32	**
*Chorizanthe*	−1.42	***	*-*	−0.31		0.19		−0.91	*
*Plantago*	0.644	***	*-*	−2.5	***	0.07		−1.16	*
*Salvia*	−0.34	*	*-*	−0.62	***	0.396	*	-	
*Vulpia*	0.794	***	*-*	−0.91	***	-		-	

Significance codes: 0 ‘***’ 0.001 ‘**’ 0.01 ‘*’ 0.05 ‘.’ 0.1 ‘ ’ 1.

The intercept represents the mean for the baseline scenario of thatch removed, summer, and fungicide added. The subsequent coefficients represent the change relative to the baseline. The first row lists the parameter estimates for the GLMM with species- and bag- level random effects. The subsequent rows list the best fit quasibinomial GLM for each species separately. The dash indicates a factor that was not included in the best-fit model.

**Figure 4:**
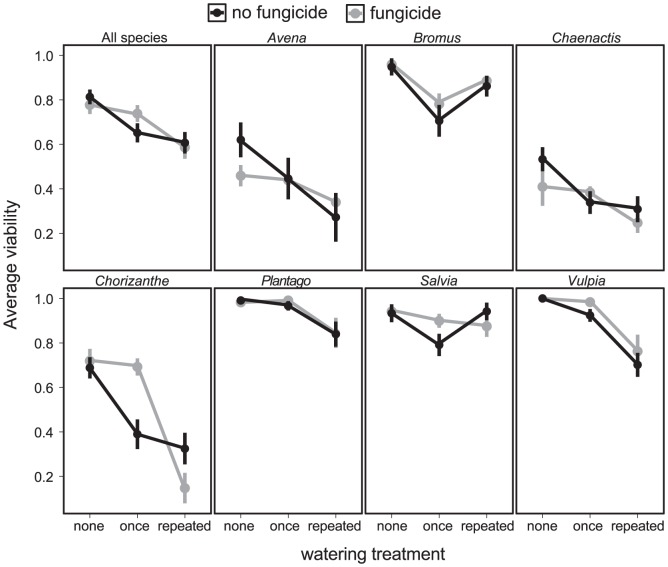
Average seed viability as a function of watering and fungicide treatments, from the laboratory experiment. The top left panel is all species combined, and the rest are individual species. Moisture treatments are dry, pulse, and wet, and fungicide treatments are with (gray points) and without (black points) fungicide. Error bars are ±1 standard error.

The laboratory experiment further confirmed the negative effect of water on seed survival and suggested a possible role of fungi in this decline. Seed survival was highest in samples that were never watered, (Figure 4; Table 4). As expected, survival declined as watering increased from never to once to continuously. However, *Bromus* and *Salvia* deviated from this pattern, having the lowest survival when watered only once (Figure 4). Fungicide slightly improved survival in samples that were watered once, but not in samples that were watered continuously (Table 4, All Species model). However, this improved survival with fungicide in samples watered once was significant only for *Chorizanthe* in the single-species models (Table 4), suggesting that fungal seed mortality is variable across species. Fungicide may either have killed beneficial mutualist fungi or have a toxic effect on seeds, which could explain the slight decrease in survival for *Chorizanthe* and *Salvia* with fungicide in the continuous watering treatments. Because fungicide treatments were applied only once at the beginning of the study, fungicide may have washed away in the continuous watering treatment.

**Table 4:** Estimates of regression parameters from the best fit models for the laboratory experiment.

	intercept		watered once		continuous watering		no fungicide	watered once×no fungicide		continuous watering×no fungicide
**All species combined**	2.05	***	−0.44	.	−1.49	***	0.27	−0.90	**	−0.20
***Avena***	0.15		−0.37		−0.97	**	-	-		-
*Bromus*	2.98	***	−1.93	***	−1.08	*	-	-		-
***Chaenactis***	−0.12		−0.44	.	−0.84	**	-	-		-
***Chorizanthe***	0.92	**	−0.13		−2.59	***	−0.09	−1.19	*	1.00
***Plantago***	4.31	***	−0.48		−2.69	**	-	-		-
*Salvia*	2.63	***	−0.93	.	−0.35		-	-		-
***Vulpia***	21.75	***	−18.73	***	−20.76	***	-	-		-

Significance codes: 0 ‘***’ 0.001 ‘**’ 0.01 ‘*’ 0.05 ‘.’ 0.1 ‘ ’ 1.

The intercept represents the mean for the baseline scenario of never watered with fungicide added. The subsequent parameters represent the change relative to the baseline. The first row lists the parameter estimates for the GLMM with species- and observation- level random effects. The subsequent rows list the best fit quasi-binomial GLM for each species separately. The dash indicates a factor that was not included in the best-fit model. Species listed in bold followed the survival pattern never watered > watered once > continuous watering.

## Discussion

In this study, I investigated patterns and drivers of seed survival in California annual plant communities with a field survey, a field experiment, and a laboratory experiment and fifteen species. The most notable result was the variation in seed survival across species, location, and season. Soil moisture was related to seed survival in all three experiments. In the year-long survey of seed survival across coastal and inland grasslands and coastal sage scrub habitats, the interaction between soil moisture and geographic location explained significant variation in survival for most species (Table 2; Figure 2). Some of the effect of soil moisture on seed survival may be mediated by soil fungi. In the field experiment at one of the survey sites, fungicide application modestly improved survival for most species in the wet winter months but not in the dry summer (Table 3; Figure 3). Similarly, seed survival in the laboratory declined in treatments that received water either once or throughout the experiment (Table 4; Figure 4). Fungicide improved survival in watered treatments in some cases, but the effect was not consistent across species (Table 4; Figure 4). Taken together, these experiments provide evidence for soil moisture-mediated seed survival, which may be partly due to soil-borne fungi. These results agree with the findings of previous studies in which seed survival varied by species, soil moisture, and habitat type, with part of these effects due to fungal pathogens [10], [14].

Although species varied considerably in seed survival and responses to environmental drivers, soil moisture appeared to play a role in all three studies. That species differ in seed survival and in responses to moisture and fungi is not surprising given the range of seed characteristics (e.g. size, shape, seed coat) as well as the range of specific and non-specific soil pathogens that naturally vary across the landscape. Although the effect of fungicide was relatively weak and variable across species, the estimated effect sizes are conservative given that a single dose of non-specific fungicide was applied only once in both of the experiments. In particular, in the winter trial in the field experiment and the wet treatments in the lab experiment some of the fungicide probably washed away. In addition, the fungicide may have killed beneficial fungi such as mychorrizae, so the fungicide treatments demonstrate the net effect of fungi in the system. This study also does not account for other water-dependent pathogens in the soil such as bacteria and *Pythium* species, which could affect seed survival.

The field survey did not support a strong role of vegetation type in mediating seed survival, although there was a significant effect of vegetation type for three species. This suggests that patterns of season- and fungal-mediated seed survival are usually a function of the seed species, not the surrounding vegetation. Additionally, vegetation types (e.g. grasslands versus coastal sage scrub) do not appear to affect seed survival consistently, for example by promoting fungal growth on all seeds. Instead, species responded idiosyncratically to vegetation type, geographic location, and, to some extent, soil moisture.

Although consistent with the results of the field and laboratory experiments, the field survey provides only a snapshot of the relationship between soil moisture and seed survival taken at one point during the rainy season (not within two weeks of a rain event). Because soil moisture was measured near its peak, this may reflect the time of year when soil moisture has the strongest effect on survival. Unmeasured aspects of the soil moisture regime such as differences in summer soil moisture may also affect seed survival. Some of the residual variation in survival across locations may be due to unmeasured variation in soil moisture.

In the field survey, I expected coastal locations to be wetter and therefore have lower seed survival, but soil moisture was actually lower in coastal locations, despite foggier and more humid conditions. This was likely due to the differences in soil type. Coastal soils were looser and more sandy, whereas inland soils contained more clay and held more water. These soil type differences may partly explain the steeper decline in seed survival with soil moisture at inland locations.

This study showed considerable variation in seed survival across species, with some evidence for a soil moisture- mediated effect of fungal pathogens. A more comprehensive pathogen removal study that also manipulated soil moisture in the field would greatly clarify the role of moisture-mediated fungal pathogens in seed survival. Fungicide could be applied repeatedly throughout the year while controlling for the effects on soil moisture using water-only controls. Sterilization of field soils (e.g. by autoclaving) would elucidate the importance of the soil biome as a whole, including fungi, bacteria, nematodes, and other organisms. Finally, direct manipulation of soil moisture in the field across geographic locations (ideally crossed with a fungicide manipulation) would clarify the role of soil moisture and its impact on pathogenic fungi. However, given the variability among species, site, and season in this study, researchers interested in controlling seed banks for particular locations or species will likely need to study species-specific drivers of seed survival at a local scale.

Seed survival is important for plant species in variable environments, such as the Mediterranean climate in southern California, where rainfall can vary six-fold between years [22]. Banking ungerminated seeds in the soil is a bet hedging mechanism that aids population recovery following years of poor seed production due to competition or environmental factors [23]. Seed banking is also important for some mechanisms of species diversity maintenance that rely on species partitioning the environment through space and time [24], [25]. Understanding the processes that control seed survival as well as the variability in seed survival across species, space, and time is important for understanding these population and community level effects of seed banking. Nonetheless, to date, ecologists are only beginning to understand the types of habitats in which species can efficiently bank seeds. This study supports the notion that ecologists might expect wetter sites to have generally lower seed survival, and that fungal pathogens are likely contributors to this effect. On the other hand, this study suggests that arid sites and drought years may have longer-lived seed banks.
